# Dental Informatics to Characterize Patients with Dentofacial Deformities

**DOI:** 10.1371/journal.pone.0067862

**Published:** 2013-08-05

**Authors:** Seoung Bum Kim, Jung Woo Lee, Sin Young Kim, Deok Won Lee

**Affiliations:** 1 School of Industrial Management Engineering, Korea University, Anam-Dong, Seongbuk-Gu, Seoul, Republic of Korea; 2 Department of Oral and Maxillofacial Surgery, Kyung Hee University, Sangil-Dong, Gandong-Gu, Seoul, Republic of Korea; Wayne State University, United States of America

## Abstract

Relevant statistical modeling and analysis of dental data can improve diagnostic and treatment procedures. The purpose of this study is to demonstrate the use of various data mining algorithms to characterize patients with dentofacial deformities. A total of 72 patients with skeletal malocclusions who had completed orthodontic and orthognathic surgical treatments were examined. Each patient was characterized by 22 measurements related to dentofacial deformities. Clustering analysis and visualization grouped the patients into three different patterns of dentofacial deformities. A feature selection approach based on a false discovery rate was used to identify a subset of 22 measurements important in categorizing these three clusters. Finally, classification was performed to evaluate the quality of the measurements selected by the feature selection approach. The results showed that feature selection improved classification accuracy while simultaneously determining which measurements were relevant.

## Introduction

Dental health is one of the most important factors in our lives. Although the advent of high information technology and dental devices has produced vast amounts of data, relatively little research has been conducted to retrieve meaningful information from dental data. However, this has been changing with the development of informatics that allows acquisition of relevant information to guide dental treatment increasingly becomes an important scientific discipline [1].

Among the various procedures that lend themselves to such data mining, orthodontic treatment of malocclusion patients to correct the position of teeth and improve appearance is well suited to use these techniques. Various analysis and simulators have been used to help dentists properly diagnosis and predict the outcome of intervention before actual treatment. Downs introduced Downs' analysis, the first systematized analytic diagnostic procedure for the roentgenographic assessment of craniofacial, skeletal, and dental patterns [2]. Down's analysis has been used by many orthodontists and by oral and maxillofacial surgeons. Based on the location of anatomical landmarks, various lengths and angles can be measured and compared with normal ranges [3], [4]. However, the most commonly used analysis is the Steiner analysis that can provide guidelines for planning of treatment based on the prediction of changes that will occur as the result of growth and orthodontic therapy [5]. The Sassouni Cephalometric Analysis has been also beneficial to dentists in functional orthodontic treatment of TMD (temporomandibular disorders) patients [6], [7]. This analysis is especially useful for determining the growth potential of these patients and in determining vertical proportions [8], [9]. Wits analysis for the diagnosis of anteroposterior discrepancy was first described by [10]. McNamara's Analysis combines the anterior reference plane (a plane perpendicular to the Frankfort horizontal through the nasion) described by Burstone et al. [11], [12]. McNamara's analysis is suitable to diagnosis, treatment planning, and treatment evaluation for not only conventional orthodontic patients, but also for patients with dentofacial deformities [13].

Although all of the a fore mentioned analyses, based mostly on simple skeletal analysis, can be useful in situations for which they were designed, prediction of postoperative outcomes nevertheless remains difficult. Despite the great potential of data mining algorithms for addressing a variety of problems in dental treatments, few efforts have been made to apply these techniques. Raberin et al. used a *k*-means clustering method with 278 dental casts of untreated French adults with normal occlusions to determine the main mandibular dental arch forms [14]. Similarly, Lee et al. used the same methodology with dental casts of 307 Korean subjects with normal occlusion to establish normative data on tooth size [15]. Hwang et al. employed a *k*-means clustering analysis to group 100 patients with facial asymmetry into five groups with different characteristics [16]. De Veld et al. detected oral cancer by applying a *k*-means clustering analysis and principal component analysis to the spectra obtained from autofluorescence spectroscopy [17].

The main purpose of the present study is to use data mining algorithms to characterize patients with dentofacial deformities. More precisely, we used a *k*-means clustering algorithm and principal component analysis to detect meaningful groups based on a number of measurements related to dentofacial deformities. Further, we used the features selection algorithm to identify which of these measurements are most important in distinguishing between the different clusters. Finally, we verified the quality of the measurements identified by the feature selection algorithm.

### Data

**Figure 1:**
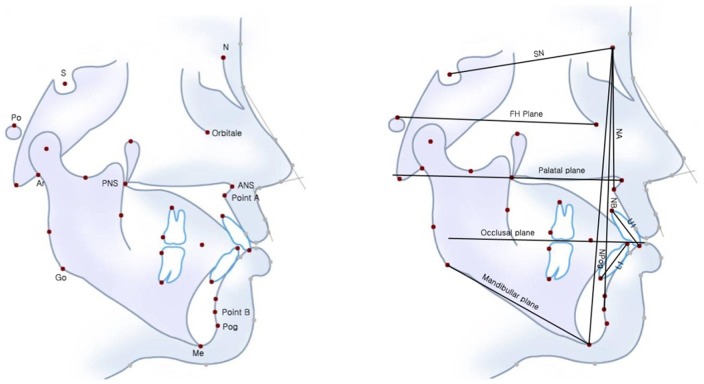
Landmark points (left figure) and planes (right figure) of lateral cephalometric radiograph.

The procedures followed were in accordance with the ethical standards and approval of the Kyunghee University Institutional Review Board (KHNMC IRB 2012–089). The participants provided their written consent to participate in this study. A total of 72 patients with skeletal malocclusions who had finished the orthodontic and orthognathic surgical treatments were enrolled for data acquisition in this study. All patients had various dentofacial deformities that required single or double jaw orthognathic correction. These deformities included maxillary horizontal hypoplasia, maxillary horizontal hyperplasia, maxillary vertical hypoplasia, maxillary vertical hyperplasia, mandibular hypoplasia, mandibular hyperplasia, and facial asymmetry. A digital panoramic and cephalometric system (Eastman Kodak Co., Rochester, New York, USA) was used to obtain various landmarks and planes that characterized the size and relationships of the teeth, jaws, and cranium. Figure 1 shows landmark points and planes that generate 22 measurements related to dentofacial deformities.

These 22 measurements can be summarized as follows:

1. **SN to FH**: An angle between the sella-nasion (SN) line and the Frankfort horizontal (FH). The SN is a line connecting the sella to the nasion. The FH is a horizontal line connecting the cephalometric porion and orbital landmarks.
2. **SN to PP**: An angle between the SN line and palatal plane (PP). PP is a line joining the posterior nasal spine and anterior nasal spline.
3. **SN to mandibular**: An angle between the SN line and mandibular plane (MP). The MP is a line/plane connecting the gonion and menton, representing the inferior border of the mandible in the sagittal plane. The mandibular plane may also be drawn as a tangent to the interior border of the mandible.
4. **FH to occlusal**: An angle between the FH and occlusal planes (OP). The OP is a line on the cephalometric radiograph representing an imaginary plane at the level of the dental occlusion.
5. **FH to mandibular**: An angle between the FH and mandibular planes (MP). The FH is a horizontal line connecting the cephalometric porion and orbital landmarks. MP is a line/plane connecting the gonion and menton, representing the inferior border of the mandible in the sagittal plane. The mandibular plane may also be drawn as a tangent to the interior border of the mandible.
6. **SNA**: An angle made up of three points: sella, nasion and point A. Point A (or ss, subspinale) is the point at the deepest midline concavity on the maxilla between the anterior nasal spine and prosthion.
7. **FH to NA**: An angle between the FH plane and the NA line.
8. **Convexity**: A distance from point A to the N-Pog line. The N-Pog line, also called the facial plane, is a line connecting the nasion and the pogonion.
9. **SNB**: An angle composed of three points: sella, nasion, and point B. Point B is the point at the deepest midline concavity on the mandibular symphysis between the infradentale and the pogonion (unilateral).
10. **SNPog**: An angle composed of three points: sella, nasion, and pogonion. The pogonion is the point of tangency of a perpendicular from the mandibular plane to the most prominent convexity of the mandibular symphysis.
11. **FH to NB**: An angle between the FH plane and the NB line. The NB is a line connecting the nasion and point B.
12. **Facial angle**: An inferior inside angle between the FH plane and the N-Pog line.
13. **Y axis**: An acute angle between the FH plane and the S-gnathion line.
14. **Gonial angle**: An angle between the mandibular plane and the posterior border of the mandible body.
15. **ANB difference**: The ANB angle (3±2°) measures the relative position of the maxilla to the mandibleThe ANB angle can be measured or calculated by the formula: ANB  =  SNA – SNB.
16. **Palatal to mandibular**: An angle between the palatal and mandibular planes.
17. **FH to U1**: The angle between the FH plane and the U1; the U1 is a line connecting the incisal edge and the root apex of the most prominent maxillary incisor.
18. **FH to L1**: The angle between the FH plane and L1; L1 is a line connecting the incisal edge and the root apex of the most prominent lower incisor.
19. **Interincisal angle**: The angle between U1 and L1.
20. **Mandibular to L1**: The angle between the mandibular plane and L1.
21. **NP to U1**: Distance from upper the incisal edge to the N-Pog line.
22. **NP to L1**: Distance from the lower incisal edge to the N-Pog line.

## Methods

### K-means clustering algorithm

We performed a clustering analysis to group 72 patients with facial deformities into several groups according to specific characteristics. Clustering analysis partitions the data by minimizing within-group variation and maximizing between-group variation [18]. These variations can be measured by various distance metrics between observations in a dataset.

In the present study we used a *k*-means clustering algorithm mainly because it is the most well-known clustering method and has been used in various applications including previous dental studies [14], [15], [16], [17]. Our procedure requires a brief summary of the *k*-means clustering algorithm. Given *k* seed points, each observation is assigned to one of the *k* seed points near the observation. This creates *k* clusters. Next, the seed points are replaced with the mean of the currently assigned clusters. This procedure is repeated with updated seed points until the assignments do not change. The results of the *k*-means clustering algorithm depend upon three parameters: distance metrics, the number of clusters (*k*), and the location of seed points.

Numerous distance metrics are available. These include the Euclidian, Manhattan, Mahalanobis, and correlation distance metrics. In the present study we relied on the widely used Euclidean distance metric. Several methods have been developed to determine the appropriate number of clusters. These include silhouette plot, gap statistics, Rand index, and adjusted Rand index methods [19], [20], [21], [22]. However, no consensus exists about which of them best satisfies all conditions. We used the Rand index and adjusted Rand index methods to determine the number of clusters. With an appropriate number *k*, the clustering algorithm that reproduces consistent clustering results would be considered the better one. The Rand index and adjusted Rand index measure the stability (i.e., consistency) of cluster results [23]. To calculate cluster stability with the Rand index and adjusted Rand index, we divided the data into three datasets. With two datasets, we conducted *k*-means clustering and got two sets of seed points. If *k* is optimal, these two sets of seed points must be similar. This means two sets of seed points with the same data should produce similar results. At this point, we have two different sets of seed points. We then split the remaining third dataset into *k* with these seed points. Finally, we used the Rand index and the adjusted Rand index to calculate cluster stability. Note that the results of both the Rand index and the adjusted Rand index lie between 0 and 1. When a cluster algorithm reproduces the same clustering results, both the Rand index and the adjusted Rand index will converge to 1 because they consider the probability of chance as the determinant of which cluster results are consistent [22]. As for determining the location of seed points, we used a random selection approach available in R software (www.r-project.org). In this study we used the “kmeans”, “randIndex”, and “adjustedRandIndex” functions in R software to implement the *k*-means clustering, Rand index, and adjusted Rand index algorithms, respectively.

### Principal component analysis

Principal component analysis (PCA) is one of the mostly widely used multivariate statistical methods for dimensionality reduction and visualization of high dimensional data [24]. PCA reduces the dimensionality of a dataset by linear combination of the original features, called principal components (PCs). Extracted PCs are uncorrelated with each other, and typically the first few PCs are sufficient to represent most of the variability in the high-dimensional original data [25], [26]. Thus, the PCA plot of observations using these first few PC axes facilitates the visualization of high-dimensional datasets. These PCs can be represented by a linear of combination of the original features (**X_1_**, **X_2_**, …, **X_p_**)
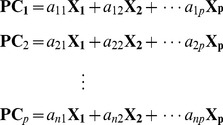
(1)


The coefficients of each PC, called loading value, can be calculated by eigenvector decomposition of the covariance (or correlation) matrix of the original data. For example, the loading values of the first PC (a_11_, a_12_, …, a_1p_) are the components of the eigenvector that corresponds to the largest eigenvalue of the covariance (or correlation) matrix. Determination of the appropriate number of PCs to retain can be subjective. Typically, a scree plot that exhibits the proportion of variance caused by each PC can be used. In a scree plot, the number of PCs to retain can be identified at an elbow point at which the proportion of variation begins to stabilize [26]. We used the “princomp” function in R software (www.r-project.org) to generate the PCA results.

### A multiple hypothesis testing procedure controlling the false discovery rate

We employed a multiple hypothesis testing procedure that controls the false discovery rate (FDR) to identify the subset of features important to distinguishing the different clusters from each other. The FDR procedure has been used to identify the significant features in high-dimensional data such as microarray, mass spectra, nuclear magnetic resonance spectra, and pairwise amino acids [27], [28], [29], [30]. First we begin with the definition of FDR, followed by the FDR procedure for feature selection. An FDR, a useful measure of the error rate in a multiple hypothesis test, is defined as the expected proportion of false positives among the all hypotheses rejected [31].

To apply FDR for feature selection, we first construct a hypothesis for each feature. More precisely, a null hypothesis, stating that the average value of the feature is equal between *k* different clusters, is established for each feature, and these hypotheses are tested simultaneously. In our study, we can construct the following multiple hypotheses for 22 features:
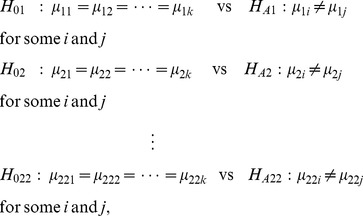
(2)where *k* is the number of clusters. Assuming that the data follow a normal distribution, we can employ an F-test for each feature by using the following test statistic:
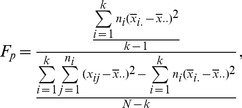
(3)for *p* = 1, 2, …, 22. 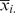 
and 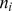
are, respectively, the sample mean and the sample size of the *i*th cluster of the *p*th feature. 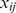
is the value for the *i*th cluster and the *j*th observation. 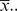
is an overall mean of the observations. Based on statistical theory, F_p_ follows an F distribution with degrees of freedom *k*-1 and *N*-*k*. Combining this with the observed F_p_ yields the *p*-value for each feature. Once we obtained a collection of *p*-values for a total of 22 features, we can use the FDR procedure that can be summarized as follows [31]:

Consider a series of *p*-values and ordered *p*-values, denoted, respectively, as *p_i_* and *p_(i),_* for *i = *1, 2,…, 22.

- Choose an FDR level 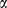 with a range between 0 and 1.
- Find 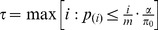
where *m* is the total number of features (here *m* = 22), 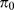 denotes the proportion of a true null hypothesis. In general, 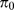 = 1 is the most conservative choice [32]. As a consequence, we used 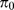 = 1.
- Let the *p*-value threshold be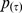. Declare the feature significant if and only if 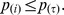

In this study we used the R software (www.r-project.org) to implement the FDR procedure.

### K-nearest Neighbors

A *k*-nearest Neighbors (KNN) algorithm is one of the most widely used algorithms for both classification and regression problems [33]. KNN does not require a trained model. Given a query point, the *k* closest points are determined. A variety of distance measures can be applied to calculate how close each point is to the query point. Then the *k-*nearest points are examined to find which of the most categories belong to the *k*-nearest points [33]. In the present study we used a KNN algorithm to computationally evaluate the features selected by an FDR procedure. We used the “knn” function in R software (www.r-project.org) to implement a KNN algorithm.

## Results

### Clustering of patients with facial deformities

The *k*-means clustering algorithm using Euclidean distance was conducted on 72 patients with facial deformities. In order to determine the appropriate number *k*, we used the Rand index and adjusted Rand index approaches described in Section 3.1. Table 1 shows the resulting Rand index and adjusted Rand index for different *k* (*k* = 2, 3, 4, 5), indicating that both methods yielded large index values when *k* = 2 or 3. We thought that using *k* = 2 was too small to capture the important grouping of the data. Thus, we chose *k* = 3 for this study.

**Table 1:** Results of the Rand index and adjusted Rand index methods to determine *k*.

k	2	3	4	5
Rand index	0.92	0.92	0.74	0.73
Adjusted Rand index	0.80	0.82	0.34	0.26

The *k*-means clustering method partitioned 72 patients into three clusters in which the first, second, and third clusters contain 17, 30, and 25 patients, respectively. Table 2 shows the descriptive statistics of the 22 measurements for each cluster.

**Table 2:** Basic statistics of features (measurements) in each cluster (*n* = the number of patients).

	Feature (Measurement)	Cluster 1(*n* = 17)	Cluster 2(*n* = 30)	Cluster 3(*n* = 25)
		Mean±SD		
1	S-N to FH	9.3±3.0	8.8±3.3	8.8±2.9
2	S-N to palatal	8.1±2.9	8.6±3.3	9.6±3.1
3	S-N to mandibular	34.7±4.4	39.7±4.9	38.7±3.7
4	FH to occlusal	6.7±4.2	9.7±3.6	11.8±3.0
5	FH to mandibular	25.4±5.0	30.9±4.6	30.0±4.1
6	SNA	83.1±2.3	79.4±3.2	80.4±2.5
7	FH to NA	92.4±2.4	88.2±2.5	89.1±1.9
8	Convexity	−0.3±8.0	−4.7±6.5	8.8±4.0
9	SNB	83.2±2.5	81.3±3.2	76.0±2.6
10	SN Pog	83.2±2.7	81.7±3.2	76.4±2.6
11	FH to NB	92.5±3.1	90.0±2.7	84.8±2.0
12	Facial angle	92.5±3.4	90.5±2.9	85.1±1.9
13	Y axis	59.3±3.4	61.5±2.9	65.4±2.5
14	Gonial angle	126.5±8.1	131.5±6.4	124.2±7.1
15	ANB difference	−0.1±3.3	−1.8±2.7	4.4±1.7
16	Palatal to mandibular	26.6±5.2	31.1±4.7	29.2±4.1
17	FH to U1	122.8±9.1	116.6±7.2	113.7±9.8
18	FH to L1	59.2±6.4	63.8±6.8	52.1±4.8
19	Interincisal angle	116.4±8.7	127.2±8.7	118.4±11.6
20	Mandibular to L1	95.4±5.6	85.2±5.8	97.9±4.9
21	NP to U1 (mm)	9.1±4.3	4.8±2.5	12.1±3.1
22	NP to L1 (mm)	9.3±3.8	5.9±3.4	7.3±2.5

**Figure 2:**
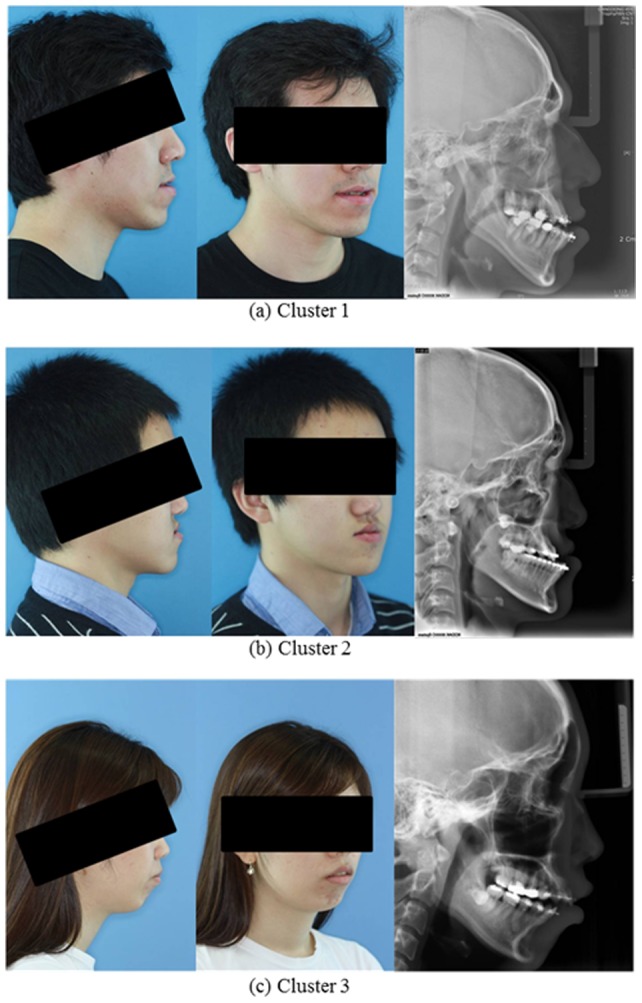
The photos and X-ray images representing three clusters identified by a k-means clustering algorithm.

Figure 2 shows the photos and X-ray images representing three clusters identified by a *k*-means clustering algorithm. The patients in the first cluster tend to have larger values of “SNB,” “SN Pog,” “FH to NB,” and “Facial angle,” but have smaller values of “FH to occlusal” and “Y axis.” In particular, “ANB difference” value is almost zero. This characteristic can be categorized into the skeletal Class III type caused by excessive antero-posterior and less vertical growth of mandible. Therefore, the patients in the first cluster require surgical treatment such as orthognathic surgery of mandible. Patients in the second cluster have smaller values of “Convexity” and “ANB difference,” but have higher values of “FH to L1” than other clusters. This is the main characteristic of the skeletal Class III type caused by the combination of maxillary deficiency and mandibular overgrowth. Consequently, these patients require bi-jaw surgery for maxillary advancement and mandibular setback. In the third cluster, the patients have larger values of “FH to occlusal,” “Convexity,” “Y axis,” and “ANB difference” than appear in other clusters. This is the main characteristic of the skeletal Class II caused by the mandibular undergrowth. Thus, the patients in the third cluster require surgical treatment for mandible advancement and genioplasty.

### Visualization of clustering results

**Figure 3:**
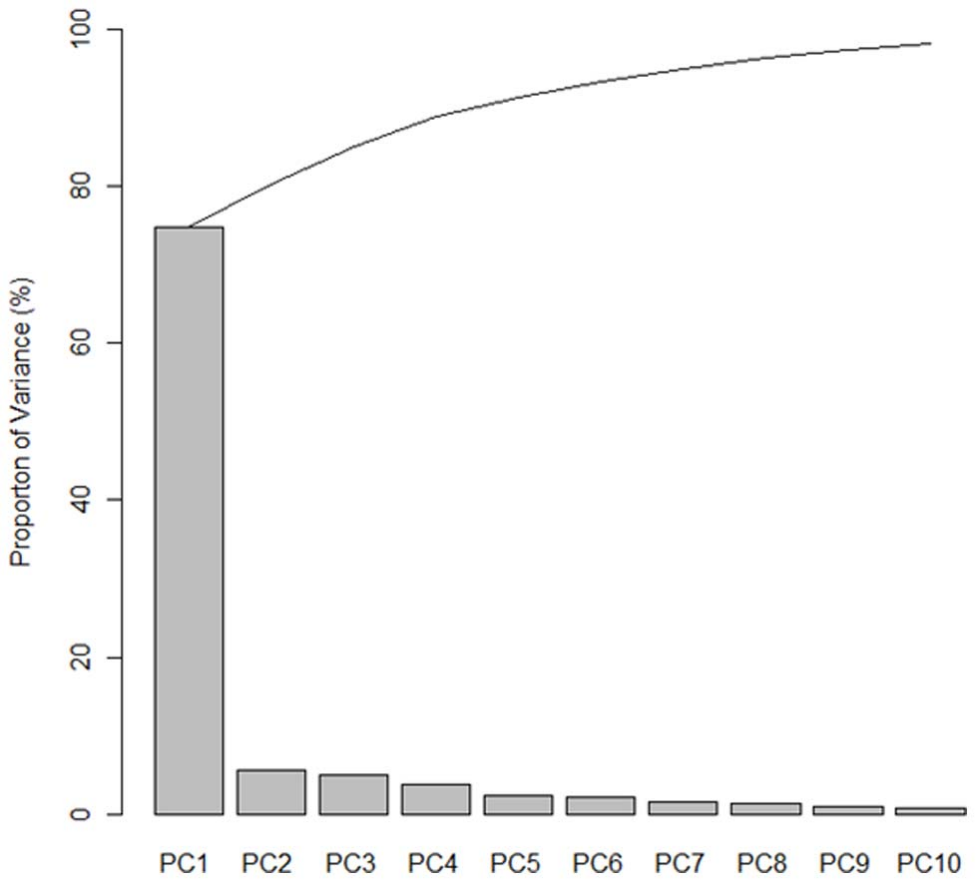
A scree plot to determine the number of PCs.

PCA can be used as a test of the validity of the groupings obtained by the *k*-means clustering analysis based on *k* = 3. The scree plot shows that the first three PC accounted for 85% of the variability of the original data (Figure 3). Thus, we used three PCs.

**Figure 4:**
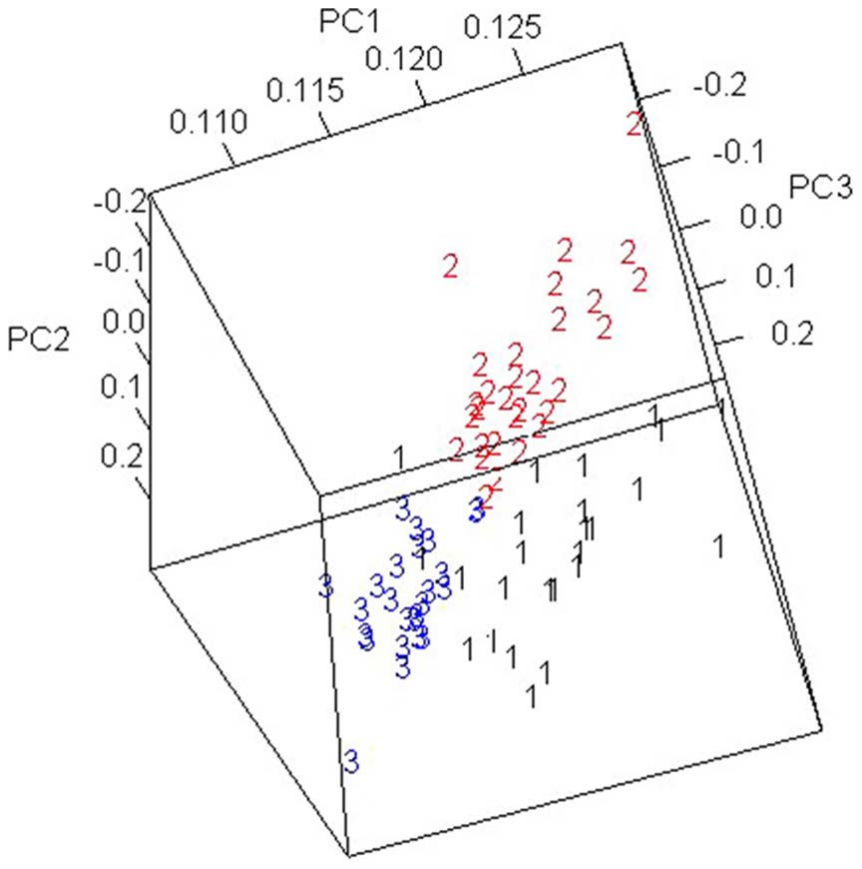
Three-dimensional PCA score plot of 72 patients with facial deformities.

Figure 4 shows a three-dimensional PCA score plot of PC1, PC2, and PC3. It clearly demonstrates that the separation of the 72 patients with facial deformities into three groups hinged on three PCs. This grouping result is consistent with the *k*-means clustering analysis.

### Identification of important features

The FDR procedure was performed to test for each feature with significant differences between the clusters at FDR levels(α) = 0.01 and 0.05. The cutoffs (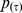) when α = 0.01 and 0.05 are 0 and 0.002, respectively.

Table 3 shows the results of feature selection using the FDR approach at α = 0.01 and at α = 0.05. Different choices of FDR levels lead to selection of different numbers of features. A higher FDR level increases the number of features selected, which results in more false positives at the same time it increases the capability to identify which features are significant. Conversely, a lower FDR level decreases the occurrence of false positives but diminishes the power to identify significant features. Here, the power is defined as the ability to correctly identify the significant features. Interpretation of our results for a case in which α = 0.05 shows that on average less than one (0.05 = 11·0.05) feature is falsely identified as significant (which is termed “false discovery”) out of the 11 features selected by the FDR procedure.

**Table 3:** Selected features by FDR procedures for α = 0.01 and 0.05.

α = 0.01		α = 0.05	
Feature	*p*-value	Feature	*p*-value
FH to occlusal	0.000	FH to occlusal	0.000
Convexity	0.000	Convexity	0.000
SNB	0.000	SNB	0.000
SN Pog	0.000	SN Pog	0.000
FH to NB	0.000	FH to NB	0.000
Facial angle	0.000	Facial angle	0.000
Y axis	0.000	Y axis	0.000
ANB difference	0.000	ANB difference	0.000
FH to L1	0.000	FH to L1	0.000
		FH to NA	0.001
		FH to U1	0.002

### Validation of the features selected

To demonstrate the validity of the feature selection results, we generated a PCA score plot using only the features selected by the FDR approach.

**Figure 5:**
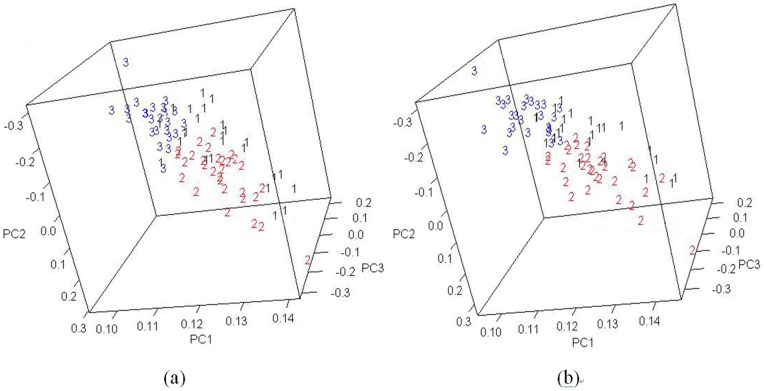
PCA plots using the features selected by (a) FDR level = 0.01 and (b) FDR level  = 0.05.

Figure 5 demonstrates that the PCA score plots produced by using the features selected by the FDR approach yielded results almost as good as the visualization capability created by using all features. This indicates that the FDR-based feature selection approach reduced the number of features required without degrading clustering performance.

The classification model is another approach to evaluating feature selection. In the present study we employed a KNN algorithm. We used Euclidean distance to determine the neighborhoods and tested different values of *k* (2, 4, 8, 16). To ensure classification accuracy, we used 80% of the dataset for training the KNN model and 20% for testing. We conducted this test 1,000 times and computed an average of 1,000 testing error rates to arrive at the final testing error rate. The datasets with different numbers of features were used for the KNN algorithm. First, we used the full dataset containing all the features. In our second and third tests we used the datasets containing the 11 and 9 features identified by the FDR approaches using α = 0.05 and α = 0.01, respectively. Table 4 shows the misclassification rates from KNN (*k* = 2, 4, 8, 16) with different numbers of features.

**Table 4:** Misclassification rate of KNN (*k* = 2, 4, 8, 16) for the datasets used with different numbers of features.

	KNN(*k* = 2)	KNN(*k* = 4)	KNN(*k* = 8)	KNN(*k* = 16)
All Features	0.22(0.10)	**0.19**(0.10)	0.20(0.11)	0.25(0.12)
FDR(α = 0.05)	0.23(0.10)	0.19(0.10)	**0.17**(0.10)	0.20(0.11)
FDR(α = 0.01)	0.23(0.10)	0.19(0.10)	**0.18**(0.10)	0.21(0.10)

Average standard errors from 1,000 experiments are shown inside the parentheses; boldface values indicate in each dataset the KNN models with minimum error rates.

This table shows that misclassification error rates are comparable for all three datasets, indicating that the subsets of features identified by the FDR-based feature selection approach achieve as good misclassification rates as methods that use all features. In conclusion, the FDR-based feature selection approach reduced the dimensionality of the original data without deteriorating classification accuracy.

**Figure 6:**
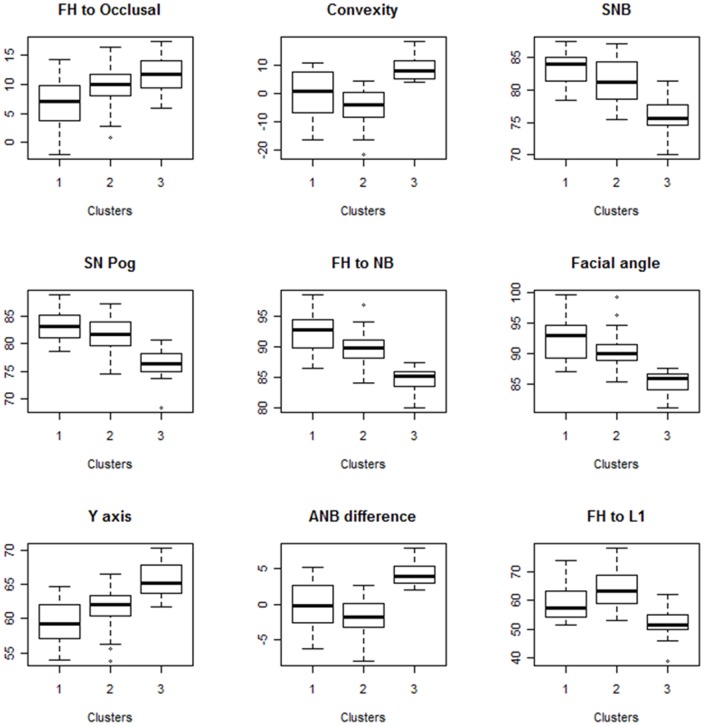
Box plots of different clusters using nine features selected by the FDR-based feature selection approach using α = 0.01.

To further explore the feature selection results (visually), Figure 6 shows the box plots of different clusters using nine features selected by the FDR-based feature selection approach using α = 0.01. We can see that at least two clusters can be distinguished by each of nine features.

## Conclusions

This paper aimed to use data mining to characterize orthodontic data. We employed a *k*-means clustering algorithm to group 72 patients with facial deformities into several groups according to their characteristics. A statistical point of view suggests that these facial deformities fit into three clusters. To investigate each cluster's characteristics, we used FDR to select the measurements important to this categorization. To interpret the validity of the results of this identification of the selected features, we used visualization and classification. PCA shows that the selected measurements yield good visualization ability by using all measurements. KNN results suggest that use of FDR reduced the dimensions involved without loss of information. These results imply that the selected features are potentially useful for understanding the pattern of facial deformities.

We believe the selected features will be a great help in diagnosis. We hope that the present study increases awareness within the dental community of efficient methodologies to improve predictive diagnosis of dental treatment.
